# Fish oil supplementation and insulin sensitivity: a systematic review and meta-analysis

**DOI:** 10.1186/s12944-017-0528-0

**Published:** 2017-07-03

**Authors:** Huanqing Gao, Tingting Geng, Tao Huang, Qinghua Zhao

**Affiliations:** 1 Centre for Lipid Research, Key Laboratory of Molecular Biology on Infectious Diseases, Ministry of Education, Chongqing Medical University, Chongqing, People’s Republic of China; 2 Epidemiology Domain, Saw Swee Hock School of Public Health, Singapore, Singapore; 3 Department of Medicine, Yong Loo Lin School of Medicine, National University of Singapore, MD1, 12 Science Drive 2, #09-01T, Singapore, 117549 Singapore; 4 Department of Nursing, The First Affiliated Hospital of Chongqing Medical University, No. 1 Youyi Lu, Yuzhong District, 400016 Chongqing, People’s Republic of China

**Keywords:** Fish oil, Insulin sensitivity, Omega-3 polyunsaturated fatty acids, Meta-analysis

## Abstract

**Background:**

Fish oil supplementation has been shown to be associated with a lower risk of metabolic syndrome and benefit a wide range of chronic diseases, such as cardiovascular disease, type 2 diabetes and several types of cancers. However, the evidence of fish oil supplementation on glucose metabolism and insulin sensitivity is still controversial. This meta-analysis summarized the exist evidence of the relationship between fish oil supplementation and insulin sensitivity and aimed to evaluate whether fish oil supplementation could improve insulin sensitivity.

**Methods:**

We searched the Cochrane Library, PubMed, Embase database for the relevant studies update to Dec 2016. Two researchers screened the literature independently by the selection and exclusion criteria. Studies were pooled using random effect models to estimate a pooled SMD and corresponding 95% CI. This meta-analysis was performed by Stata 13.1 software.

**Results:**

A total of 17 studies with 672 participants were included in this meta-analysis study after screening from 498 published articles found after the initial search. In a pooled analysis, fish oil supplementation had no effects on insulin sensitivity compared with the placebo (SMD 0.17, 95%CI -0.15 to 0.48, *p* = 0.292). In subgroup analysis, fish oil supplementation could benefit insulin sensitivity among people who were experiencing at least one symptom of metabolic disorders (SMD 0.53, 95% CI 0.17 to 0.88, *p* < 0.001). Similarly, there were no significant differences between subgroups of methods of insulin sensitivity, doses of omega-3 polyunsaturated fatty acids (n-3 PUFA) of fish oil supplementation or duration of the intervention. The sensitivity analysis indicated that the results were robust.

**Conclusions:**

Short-term fish oil supplementation is associated with increasing the insulin sensitivity among those people with metabolic disorders.

## Background

Type 2 diabetes mellitus (T2DM) is one of the most common chronic diseases caused a dramatic public health burden overall and T2DM is also linked to a higher risk of all types cancer incidence, which is reported to be one of the leading causes of morbidity and mortality worldly [1]. It is estimated that the prevalence of T2DM will increase to 4.4% in 2030 and the total number of all age-group people is projected to rise to over 250 million by then [2]. The direct economic burden related to T2DM, gestational diabetes and impaired glucose tolerance exceeded over $ 300 billion in 2012 in the USA [3]. It has been systematically reported that fatty acids have great influences on a series of chronic diseases, such as T2DM, cardiovascular diseases and metabolic syndrome [4–7]. Previous meta-analysis studies have reported that fish consumption and dietary long-chain omega-3 polyunsaturated fatty acids (n-3 PUFA) decreased the risk of T2DM [8, 9]. The active elements of fish oil supplementation are recognized as n-3 PUFA (DHA and EPA) [4, 5]. In this study, to reduce the bias, only fish-derived n-3 PUFA was included rather than the plant-derived intervention studies.

Insulin sensitivity is a measure of insulin responsiveness. Poor insulin sensitivity is associated with T2DM and metabolic syndrome [10, 11]. The results of randomized control trials (RCTs) investigating the association between n-3 PUFA and insulin sensitivity were inconsistent [12–15]. The different results may be caused by the various study population, small sample size, short study duration, different dose of n-3 PUFA intervention. In 2011, Akinkuolie et al. conducted a meta-analysis about the relationship between n-3 PUFA and insulin sensitivity [16], which reported that n-3 PUFA had no effects on insulin sensitivity. However there have been new RCTs published after this meta-analysis above. Therefore, we conducted a comprehensive and updated meta-analysis to systematically examine the effect of fish oil supplementation on insulin sensitivity.

## Methods

### Search strategy

The database of Cochrane, PubMed, and Embase up to Dec 2016 were searched and this report was performed according to the Preferred Reporting Items for Systematic Reviews and Meta-Analysis (PRISMA) statement [17]. The search was based on combinations of synonyms for fish oil (omega-3 fatty acid, n-3 fatty acid, DHA, EPA), insulin resistance and insulin sensitivity. References lists of retrieved articles were screened manually to achieve maximum sensitivity of the search strategy and identify all randomized control trials (RCTs). The measures of insulin sensitivity estimate included of hyperinsulinemic-euglycemic glucose clamp, HOMA of insulin resistance (HOMA-IR), Quantitative Insulin Sensitivity Check Index (QUICKI) and glucose tolerance.

### Search selection

RCTs (either parallel or crossover design) were selected by two authors (TT G, HQ G) following the inclusion criteria: human RCTs with fish oil supplementation intervention, including of omega-3 fatty acid, n-3 fatty acid, DHA, EPA, and the outcomes were values of baseline and after intervention insulin sensitivity or insulin resistance. Data of insulin sensitivity estimates should be sufficient to calculate mean and standard deviation (SD). The participants of studies were adults, who were over 18 years old. The control group could receive corn oil, olive oil or other types of placebos. However, the placebo could not be any capsule containing n-3 fatty acid elements. The studies that target populations were children or adolescents under 18 years old were excluded. Previous studies have demonstrated that physical activity could increase in insulin sensitivity [18, 19]. We also excluded the studies that combined fish oil supplementation with other physical activities as an intervention. We also excluded the studies that combined fish oil supplementation with other physical activities as an intervention. Nonclinical trials, observational studies or reviews were excluded. To avoid the carry-over effect, all the crossover design studies were extracted the data from the first period only, managed as parallel group trials. Therefore, crossover design studies without specific data at the end of the first intervention phase were excluded [20]. Only English trials were included in this study. A senior author (QH Z or TH) was consulted when there were discrepancies about the study inclusion.

### Data extraction and quality assessment

A standard data extraction form was used by two authors independently to collect the Information, which included name of the first author, publication year, study design, inclusion/exclusion criteria, sample size, participants’ characteristics, intervention details (e.g. dosage, type, frequency, duration), placebo, the outcomes information (e.g. Insulin sensitivity, insulin resistance) and compliance. Sample size, mean and SD were extracted from intervention group and control group. Only the first duration data were obtained from the cross-over design studies. Two researchers (TT G, HQ G) assessed the methodological quality of each trial included in the study independently. When there were disagreements of the data extraction, a third author (QH Z or TH) was consulted. Cochrane Collaboration’s tool was used to assess the quality of the trials, which included of randomization procedures, allocation concealment, blinding of participants, researchers and outcome assessors, incomplete outcome data, non-elective reporting, other bias and compliance. If all features were adequate, the quality of the studies was a low risk of bias. If one or more features were unclear, the risk of bias was unclear. If one or more features were inadequate or negative, it was at high risk of bias.

### Data synthesis and analysis

The mean and SD values of intervention group and control group at the end of the fish oil supplementation intervention were extracted to calculate the effect size [21]. If SEs were reported rather than SDs, then SDs were calculated by equation $\mathrm{SD}=\mathrm{SE}\times \sqrt[2]{n}$. If 95% CI was reported, SD was calculated by equation $\mathrm{SD}=\sqrt[2]{n}\times \left(\mathrm{upper}-\mathrm{lower}\right)/2\times \mathrm{t}$, where n is the number of subjects [22]. A random-effects model (using the DerSimonian-Laird method) and the generic inverse variance method were used to derive pooled estimates across studies [23]. The heterogeneity was assessed through the I^2^ statistic and *p*-value. I^2^ > 50% was considered as the substantial amount of statistical heterogeneity [24]. The publication bias was evaluated by Egger’s and Begg’s method. Sensitivity analysis was conducted when heterogeneity was more than 50% combined *p*-value less than 0.05. Influence analysis method was used to perform the sensitivity test. Omitting one study once till all the studies were picked out during the sensibility test. The studies, which affected the results primarily to cause heterogeneity, were excluded. In order to assess the potential sources of heterogeneity, subgroup analysis were designed to conduct according to participants’ characteristics (healthy persons, metabolic disorders participants, T2DM), measurement of insulin sensitivity (clamps, HOMA, QUICKI, glucose tolerance), n-3 PUFA dose (low dose < 2 g, high dose ≥ 2 g) [25], intervention duration (short-term < 12w, long-term ≥ 12w) [26]. All tests were performed using statistical software package STATA version 13.1 and two-sided *P* value <0.05 were considered statistically significant.

## Results

### Study identification

**Fig. 1:**
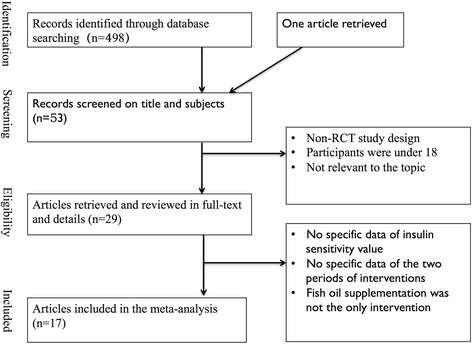
Summary of the procedure used to select studies for inclusion in the meta-analysis

Seventeen studies included in this meta-analysis after the screening involving 672 participants. 498 published articles were found after the initial search. One article retrieved from hand search through references of included studies. After the titles and abstracts screening, there were 53 articles left. Studies were excluded by non-RCTs study design or not relevant to the topic, or the participants were under 18 years old. Studies were excluded due to the fish oil supplementation was not the only intervention or there were no specific data of the insulin sensitivity estimates. Crossover design studies were excluded without the specific data of the first period of interventions. After the full-text screening, 17 trails met the criteria and were assessed the study quality. The details of the studies selection process are shown in Fig. 1.

### Study characteristics

Descriptive data of the included studies were summarized in Table 1. All the included trials were published from 1995 to 2014. One of the 17 studies was crossover design [27] and others were parallel design. The participants from 8 studies included were diagnosed as type 2 diabetes [14, 15, 28–30] and participants of 4 studies were healthy persons [27, 31, 32]. Others were patients with at least one of the metabolic disorder symptoms [12, 13, 33–35], including of abnormal boday mass index (BMI), hypertension, and lipid metabolism disorders. Four of the included studies used clamps as the methods of insulin sensitivity estimates [15, 28, 29]. HOMA [12–14, 27, 30, 34, 36, 37] and QUICKI [31, 38, 39] were used by 9 studies and 3 trials, respectively. One of the trials used glucose tolerance method [33]. The doses of active ingredients of fish oil (n-3 fatty acids) were ranged from 1 g/d to 4 g/d. Duration of the interventions was ranged from 4 weeks to 24 weeks. All the trials have reported the baseline values of insulin sensitivity and the data of insulin sensitivity after interventions.

**Table 1:** The characteristic of the RCTs included in our study

Study (year)	Population characteristics	Study design	Age (years old)	Gender	n-3PUFA dose	Duration	Control	IS measure	Compliance
Rivellese 1996 [28]	16 postmenopausal women with NIDDM and type 2B or IV hyperlipoproteinemia	RCT	40–75	F = 16	2.5 g in the first 2 mo; 1.7 g in the last 4 mo	24w	Olive oil	Euglycemic hyperinsulinemic clamps	Acceptable
Krysiak 2011 [12]	66 Patients with lipid metabolism abnormalities	RCT	35–70	M = 43 F = 23	2 g/d	12w	Placebo	HOMA ratio	90%–100%
Rizza 2009 [13]	50 healthy patients with ≥ 1 parent with DM2	RCT	29.9 ± 6.2	M = 25 F = 25	2 g/d	12w	Olive oil	QUICKI	93%
Woodman 2002 [14]	59 T2DM with oral hypoglycemic medications	RCT	40–75	M = 39 F = 12	4 g/d	6w	Olive oil	Euglycemic hyperinsulinemic clamps	98%
Mostad 2006 [15]	26 T2DM without hypertriacylglycerolemia	RCT	40–75	M = 13 F = 14	2.4 g/d	9w	Corn oil	Isoglycemic hyperinsulinemic clamps	96%
Sarbolouki 2013 [30]	67 patients with T2DM	RCT	35–55	M = 26 F = 41	2 g/d	12w	Corn oil	HOMA-IR	96%
Morvarid 2007	26 postmenopausal women with T2DM	RCT	40–60	F = 26	3 g/d	8w	Paraffin oil	HOMA-S	90%
Darshan 2011	34 healthy men	RCT	39–66	M = 34	3 g/d	12w	Olive oil	HOMA-IR	85%
Fakhrzadeh 2010 [39]	134 elderly T2DM	RCT	≥65	F = 79 M = 55	1 g/d	24w	Medium chain triglycerides oil	HOMA-IR	93%
Michael 2013	34 non diabetic subjects with either impaired glucose tolerance impaired fasting glucose, or at least three features of the metabolic syndrome	RCT	27–43	M = 12 F = 22	4 g/d	12w	Corn oil	Intravenous glucose tolerance tests	97%
Abete 2008 [35]	32 subjects (BMI = 31.6 ± 3.5)	RCT	36 ± 7	F = 14 M = 18	1.69 g/d	8w	Control diet	HOMA index	85%
Farsi 2013	45 patients with T2DM	RCT	30–65	NA	4 g/d	10w	Corn oil	QUICKI	98%
Toft 1995 [48]	78 persons with untreated hypertension	RCT	20–61	M = 50 F = 28	4 g/d	16w	Corn oil	Euglycemic hyperinsulinemic clamp	96%
Crochemore 2012 [37]	27 women with high blood pressure and T2DM	RCT	60.64 ± 7.82	F = 27	2.5 g/d	4w	Gelatin	QUICKI	100%
Kondo 2014 [26]	23 postmenopausal women	RCT	69.7 ± 6.6	F = 23	≥3 g	4w	Fish avoid intake diet	HOMA-R	100%

### Quality assessment

The Cochrane Collaboration’s tool for assessing the risk of bias included the adequate sequence generation, allocation concealment, blinding, incomplete outcome data, selective reporting and another risk of bias. All the trials rarely stated about the specific process of allocation concealment. Two studies were not double blinded properly and one study was single blinded. Two were not clear about the blinding. One of the studies did not report the exact information of the loss to follow-up and did not explain in detail either. Details of the quality assessment could be seen in Table 2.

**Table 2:** Quality assessment of the included studies

Study	Random sequence generation	Allocation concealment	Blinding of participants	Blinding of outcome	Incomplete outcome data addressed	Non-elective reporting	Other bias
Rivellese 1996 [28]	U	U	L	L	L	L	L
Krysiak 2011 [12]	U	U	U	U	L	L	U
Rizza 2009 [13]	U	U	L	L	L	L	L
Woodman 2002 [14]	U	L	L	L	L	L	L
Mostad 2006 [15]	U	U	L	L	L	L	L
Sarbolouki 2013 [30]	U	U	L	L	L	L	L
Morvarid 2007	U	U	L	L	L	L	L
Darshan 2011	U	U	U	U	L	L	U
Fakhrzadeh 2010 [39]	U	U	L	L	L	L	L
Michael 2013	U	U	U	U	U	U	U
Abete2008 [35]	U	U	U	U	L	L	U
Farsi 2013	U	U	U	U	L	L	U
Toft 1995 [48]	U	U	L	L	L	L	L
Crochemore 2012 [37]	U	U	L	H	L	L	H
Kondo 2014 [26]	U	U	H	H	L	L	H

*H* high risk, *L* low risk, *U* unclear

### Meta-analysis

**Fig. 2:**
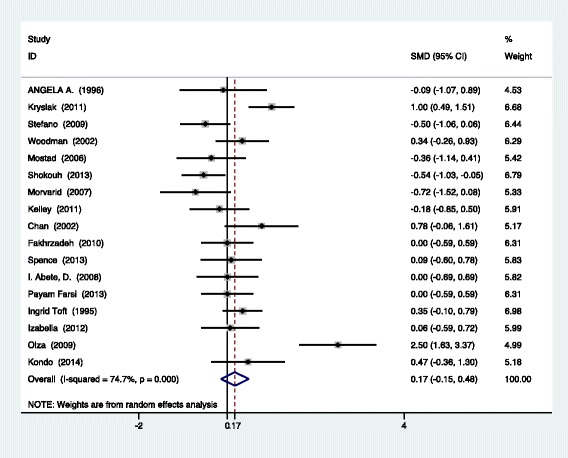
Meta-analysis of insulin sensitivity of participants with fish oil supplementation

The forest plot of insulin sensitivity is shown in Fig. 2. The pooled analysis showed that fish oil supplementation had no effects on insulin sensitivity overall (SMD 0.17, 95%CI -0.15 to 0.48, *p* = 0.292). The I^2^ test indicated that there was a substantial statistical heterogeneity across the included trials (I^2^ = 58.1%, *p* = 0.001).

### Subgroup analysis

The results of the subgroup analysis present in Table 3 showed an association of lower risk in the group of people with metabolic disorders (SMD 0.53, 95%CI 0.17–0.88, *p* < 0.001). We found fish oil had no effects on insulin sensitivity among the healthy people or people with T2DM. Also, subgroup analysis showed a positive effect of fish oil on insulin sensitivity among the short-term intervention group rather than the long-term intervention group (SMD 0.31, 95%CI 0.01–0.61, *p* = 0.04). There was no difference in the subgroups of intervention dosage and methods of insulin sensitivity values. Results of subgroup analysis are shown in Table 3.

**Table 3:** Subgroup analysis of fish oil consumption and insulin sensitivity

Subgroup	No. Of studies	SMD (95%IC)	*P* value	I^2^ value (%)
Methods of insulin sensitivity				
Clamps	4	0.10(−0.18–0.44)	0.41	0.00
HOMA	9	0.28(−0.08–0.63)	0.13	66.6
QUICKI	3	0.15(−0.68–0.97)	0.73	81.6
Glucose tolerance	1	0.19(−0.05–0.42)	0.79	58.1
Population				
T2DM	8	0.12(−0.22–0.45)	0.50	57.1
Metabolic disorders	5	0.53(0.17–0.88)	<0.001	42.6
Healthy people	4	-0.15(−0.53–0.24)	0.46	22.8
Dose				
≥2 g	14	0.17(−0.11–0.46)	0.24	64.2
< 2 g	3	0.26(−0.04–0.56)	0.09	0.00
Duration				
≥12w	9	0.09(−0.25–0.44)	0.60	70.2
<12w	8	0.31(−0.01–0.61)	0.04	29.4

### Sensitivity analysis and publication bias

In sensitivity analysis, it could be noticed that there was no any trial’s estimated value being out of the 95% CI and the combined 95% CI. The summary results did not differ significantly when we omitted studies one at a time. Therefore, results remained stable and robust. There was no suggestion of small study effect based on visual inspection of the funnel plot. Results of the Egger’s (*p* = 0.78) and Begg’s (*p* = 0.43) tests showed that there was no potential publication bias.

## Discussion

The findings from this meta-analysis, based on 672 participants from 17 studies, demonstrated that fish oil supplementation did not improve insulin sensitivity overall. In subgroup analysis, among participants with metabolic disorders, fish oil supplementation could reduce the risk of insulin resistance by 47%. Our results were consistent with the meta-analysis reported in 2011 showing that fish oil supplementation had no effects on insulin sensitivity; however, among participants with metabolic syndrome, fish oil is associated with lower risk of insulin resistance [16, 40]. Participants in the meta-analysis were only healthy people and T2DM people; therefore, an important group of patients with metabolic disorders was neglected in the research. It has reported that insulin sensitivity is strongly associated with metabolic syndrome [41]. In addition, the previous meta-analysis did not address issues relating to dose and duration of intervention. Our study made subgroup analysis according to dose and duration of intervention, though the results were negative. Observational studies have reported that high dose of n-3 PUFA were effective to prevent chronic diseases rather than low dose and high dose n-3 PUFA could reduce serum triglyceride concentrations effectively and hypertriglyceridemia [25]. However, epidemiological studies do not prove the causality of interventions on outcomes. In our meta-analysis, there was no evidence showing the difference of dose intervention of fish oil supplementation on insulin sensitivity.

Our study suggested that short-term fish oil supplementation was beneficial for insulin sensitivity than long-term intervention. Additionally, the previous study has reported that serum PUFA levels reaching equilibrium needed 4 weeks at least [26]. Combining with the results of our research, long-term treatment duration intervention was not effective on insulin sensitivity either. Therefore, the best intervention period might be short-term, which is less than 12 weeks but at least over 4 weeks. Due to the serum biomarkers’ metabolism period, duration of the intervention can be critical for the study. As the reasons mentioned, more studies are needed to explain the duration related difference. The dose of n-3 PUFA did not affect the insulin sensitivity in our research. In Akinkuolie et al.’s study, he focused on the relationship between n-3 PUFA and insulin sensitivity. Therefore, he included the studies all containing n-3 PUFA, such as fish oil supplementation, and plant-derived n-3 PUFA (flaxseeds). It is reported that the conversion of plant-derived n-3 PUFA to DHA is relatively worse than fish oil supplementation though [4]. Therefore, it would be inappropriate to combine the studies with plant-derived and fish-derived n-3 PUFA interventions together. Our study is more accurate on fish oil supplementation and insulin sensitivity, which could reduce bias.

Potential mechanisms could explain the association between fish oil supplements and insulin sensitivity. Nutraceuticals and functional food ingredients are beneficial to insulin resistance and dyslipidemia through decreasing 7a–hydroxylase, 3-hydroxy-3-methylglutaryl-CoA, very low-density lipoprotein [42]. Lee et al. also suggested that polyunsaturated fatty acid-based dietary supplements could improve biomarkers related metabolic syndrome [43]. It is well recognized that Insulin sensitivity/resistance is associated with metabolic syndrome tightly [41]. The main signs of metabolic syndrome are visceral obesity, hypertension, evaluated fasting serum triglyceride level, impaired fasting glucose, insulin resistance, or pre-diabetes. Adiposity increases the risk of diabetes and reduces insulin sensitivity through the fat tissue release inflammatory markers, insulin-like growth factors (IGF), sCD163 and adipokines [44–46]. Thus, it is biologically plausible that fish oil supplementation could improve insulin sensitivity among people with metabolic disorders.

## Strengths and limitations

This meta-analysis was an updated and comprehensive investigation on the effects of fish oil supplementation and insulin sensitivity. We included RCTs, which avoids the influence of bias of observational studies.

There are several limitations that should be acknowledged in our study. One of the limitations of this meta-analysis is the quality of methodologies across the trials included. Some of the trials were not blinded well or randomized properly. However, sensitivity analysis showed that the results maintained robust. Well-designed RCTs are needed to perform in the future. Secondly, our study did not address the issues related to the age of participants. It is reported that the change of insulin sensitivity might be along with the changes of adiposity rather than being an inevitable consequence ageing [47]. The sample size of some studies included was relatively small; whose power was less than 80%. In addition to methodological limitations of the original studies, dosages of fish oil supplementations were different within the different studies, which could affect the final results. However, subgroup analysis showed no different effects between the high dose and low dose group of fish oil on insulin sensitivity. As to the carry-over effect of the cross design studies included, we used the first periods of intervention before cross-over. Therefore, there was no carry-over influence [20, 48]. Finally, we assumed that most of the well-designed RCTs would have been published in English language journals. However, our comprehensive literature search and the results of non-publication bias showed that our meta-analysis was not driven by a selective publication of positive findings.

## Further implication

This systematic review and meta-analysis showed that fish oil supplementation did not affect estimates of insulin sensitivity overall. However, short-term fish oil supplementation could improve insulin sensitivity among patients with metabolic disorders, which could be a significant intervention as secondary prevention for the T2DM and metabolic syndrome. The subgroup analysis suggested that there was no influence of dosage of supplementation and measure of insulin sensitivity on the outcomes. However, more interventions studies especially high-quality trials are necessary to confirm the results.

## Conclusion

Take together, these findings have great implications for prevention of T2DM. The research studies in the future would be more beneficial to explicitly prescribe interventions for trials, especially the dose, frequency, administration and duration of fish oil supplementation. Additionally, more research should be done to determine the population who could benefit from the intervention.

## Abbreviations

BMI: Body mass index
PRIMA: Preferred Reporting Items for Systematic Reviews and Meta-Analysis
PUFA: Polyunsaturated fatty acids
QUICKI: Quantitative Insulin Sensitivity Check Index
RCTs: Randomized control trials
SD: Standard deviation
T2DM: Type 2 diabetes mellitus
